# Donor‐derived M2 macrophages attenuate GVHD after allogeneic hematopoietic stem cell transplantation

**DOI:** 10.1002/iid3.503

**Published:** 2021-08-19

**Authors:** Ryo Hanaki, Hidemi Toyoda, Shotaro Iwamoto, Mari Morimoto, Daisuke Nakato, Takahiro Ito, Kaori Niwa, Keishiro Amano, Ryotaro Hashizume, Isao Tawara, Masahiro Hirayama

**Affiliations:** ^1^ Department of Pediatrics Mie University Graduate School of Medicine Tsu Mie Japan; ^2^ Department of Pathology and Matrix Biology Mie University Graduate School of Medicine Tsu Mie Japan; ^3^ Department of Hematology Mie University Graduate School of Medicine Tsu Mie Japan

**Keywords:** graft‐versus‐host disease (GVHD), hematopoietic stem cell transplantation (HSCT), M1 macrophage, M2 macrophage, M‐CSF

## Abstract

**Introduction:**

Graft‐versus‐host disease (GVHD) is frequent and fatal complication following allogeneic hematopoietic stem cell transplantation (HSCT) and characteristically involves skin, gut, and liver. Macrophages promote tissue regeneration and mediate immunomodulation. Macrophages are divided into two different phenotypes, classically activated M1 (pro‐inflammatory or immune‐reactive macrophages) and alternatively activated M2 (anti‐inflammatory or immune‐suppressive macrophages). The anti‐inflammatory effect of M2 macrophage led us to test its effect in the pathophysiology of GVHD.

**Methods:**

GVHD was induced in lethally irradiated BALB/c mice. M2 macrophages derived from donor bone marrow (BM) were administered intravenously, while controls received donor BM‐mononuclear cells and splenocytes. Animals were monitored for clinical GVHD and analyzed.

**Results:**

We confirmed that administering donor BM‐derived M2 macrophages attenuated GVHD severity and prolonged survival after HSCT. Moreover, donor BM‐derived M2 macrophages significantly suppressed donor T cell proliferation by cell‐to‐cell contact in vitro.

**Conclusions:**

We showed the protective effects of donor‐derived M2 macrophages on GVHD and improved survival in a model of HSCT. Our data suggest that donor‐derived M2 macrophages offer the potential for cell‐based therapy to treat GVHD.

## Graphical abstract

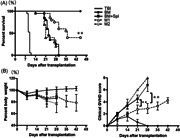

## 1 INTRODUCTION

Hematopoietic stem cell transplantation (HSCT) is a potentially curative treatment for malignant diseases as well as hematological diseases.^1^ Graft‐versus‐host disease (GVHD) is one of the major life‐threatening complications of HSCT. Although the field of HSCT has seen considerable advances in transplant procedures and pharmacotherapy, GVHD remains the second most common cause of death after disease relapse.^2^ GVHD results from donor T lymphocytes activated by recipient antigen‐presenting cells (APCs) during the initial few days after HSCT.^3^, ^4^ The conditioning regimen used during HSCT leads to tissue destruction, and this tissue damage results in excessive release of inflammatory cytokines which activates recipient APCs such as dendritic cells (DCs) and Langerhans cells. These primed APCs presenting alloantigens interact with the donor T lymphocytes.^5^, ^6^, ^7^, ^8^ Then, donor T lymphocytes undergo proliferation and differentiation and GVH reactions are induced.

Macrophages are a component of both innate and adaptive immunity and have a dual role of proinflammation and anti‐inflammation. It has been reported that macrophages can be divided into two subsets, classically activated M1 macrophages (pro‐inflammatory or immune‐reactive macrophages) and alternatively activated M2 macrophages (anti‐inflammatory or immune‐suppressive macrophages). M1 macrophages are polarized by lipopolysaccharide (LPS) in association with Th1 cytokines such as interferon (IFN)‐γ, granulocyte macrophage colony‐stimulating factor (GM‐CSF).^9^, ^10^, ^11^ They secrete tumor necrosis factor (TNF)‐α, interleukin‐1β (IL‐1β), IL‐6, IL‐12, and IL‐23.^9^, ^10^, ^11^ M2 macrophages are polarized by IL‐4 and IL‐13 and secrete anti‐inflammatory cytokines such as transforming growth factor (TGF)‐β and IL‐10.^9^, ^10^, ^11^ It has been reported that macrophage infiltration is related to occurrence and development of GVHD and a ratio of M1/M2 macrophages is associated with severity of acute GVHD.^12^, ^13^ Other studies also demonstrated that dermal macrophage infiltration is a predictive factor for refractory GVHD.^14^, ^15^ However, it is unknown whether infiltrated macrophages exacerbate or attenuate the severity of GVHD. Hashimoto et al reported that pretransplant injection of macrophage colony‐stimulating factor (M‐CSF) improves GVHD in transplant animals by host macrophage expansion.^16^ In addition, Wen et al showed that although recipients who were transplanted with bone marrow (BM) cells with low M1/M2 macrophage ratio showed a low incidence of GVHD, recipients who received BM cells with a higher M1/M2 macrophage ratio showed a higher GVHD incidence.^17^ Since anti‐inflammatory macrophages exert inhibitory effects on alloimmune responses, we speculated that M2 macrophages could attenuate severity of GVHD.

To evaluate the role of macrophage subsets for GVHD, we constructed an experimental model of HSCT from C57BL/6 (B6) donors to BALB/c recipients with or without macrophages injection. The severity of GVHD was suppressed by injection of donor BM‐derived M2 macrophages which significantly suppressed donor T cell proliferation by cell‐to‐cell contact. Therefore, our data offer promise to develop M2 macrophage cell therapy against GVHD in patients who receive HSCT.

## 2 MATERIALS AND METHODS

### 2.1 Reagents

Recombinant mouse GM‐CSF and M‐CSF cytokines were obtained from Biolegend. LPS and IFN‐γ were obtained from Sigma‐Aldrich. IL‐4 was also purchased from Biolegend.

### 2.2 Mice

Six‐week‐old female B6 (H‐2^b^), green fluorescent protein transgenic B6 (B6‐GFP, H‐2^b^), and BALB/c (H‐2^d^) mice were purchased from Japan SLC. Mice were maintained in specific pathogen‐free conditions and received autoclaved water after transplantation. All animal procedures and experiments were performed according to protocol approved by the Animal Ethics Committee (Permission number 29‐3), Mie University Graduate School of Medicine.

#### 2.2.1 Preparation of GM‐CSF‐derived and M‐CSF‐derived macrophages

BM‐derived macrophages (BMMs) induced by GM‐CSF (GM‐BMMs as M1 macrophages) and BMMs induced by M‐CSF (M‐BMMs as M2 macrophages) were prepared by a similar protocol as described previously.^18^, ^19^, ^20^, ^21^ Briefly, BM cells were obtained by flushing mouse tibiae and femurs from B6 mice with ice‐cold RPMI 1640 media (Sigma‐Aldrich) and BM mononuclear cells (BM‐MNCs) were isolated by Histopaque‐1077 (Sigma‐Aldrich). For preparation of GM‐BMMs, BM‐MNCs were seeded (10^6^ cells/ml) in RPMI 1640 medium containing 50 U/ml penicillin, 50 mg/ml streptomycin, 50 mM 2‐mercaptoethanol, 10% fetal bovine serum (FBS) and 20 ng/ml GM‐CSF or 3 days. Then, non‐adherent cells were harvested and seeded in complete medium and 20 ng/ml GM‐CSF for 4 days. Then, the medium was changed to complete medium containing 20 ng/ml of GM‐CSF, 10 μg/ml of LPS, and 10 ng/ml IFNγ, and adherent cells were incubated for more than 2 days. The adherent cells were harvested and used as GM‐BMMs for experiments. For the preparation of M‐BMMs, BM‐MNCs were cultured in complete medium containing 20 ng/ml M‐CSF for 3 days. Then, non‐adherent cells were harvested and seeded in complete medium containing 20 ng/ml M‐CSF for 4 days. Then, the medium was changed containing 20 ng/ml of M‐CSF and 10 ng/ml of IL‐4 and adherent cells were incubated for more than 2 days. The adherent cells were harvested and used as M‐BMMs for experiments.

### 2.3 RNA isolation and RT‐PCR

Total RNA was isolated from GM‐BMMs and M‐BMMs using RNeasy Mini Kit (Qiagen, Inc.) and 500 ng of total RNA was used to reverse transcribe into cDNA. Reverse transcription reactions were performed as previously described.^22^, ^23^, ^24^, ^25^ To reveal the gene expression of polarized macrophages, RT‐PCR was conducted using Takara Taq polymerase (Takara Biomedicals). The following gene‐specific primers were used: β‐actin (forward, 5ʹ‐GATGGGCAAAGGAG ATCCTAAG‐3ʹ, reverse, 5ʹ‐TCACTTTTTTGTCTCCCCTTTGGG‐3ʹ), CD11b (forward, 5ʹ‐GGCCCTTCTCCAG GACAGA‐3ʹ, reverse, 5ʹ‐GCTGATCATGGCTGGGTTGT‐3ʹ), F4/80 (forward, 5ʹ‐GTG ATGCCCCAGGCA‐3ʹ, reverse, 5ʹ‐TCTCACCCAGGGAATTCAAA‐3ʹ), CD38 (forward, 5ʹ‐TTGCAAGGGTTCTTGGAAAC‐3ʹ, reverse, 5'‐CGCTGCCTCATCTACACTC A‐3ʹ), TNF‐α (forward, 5ʹ‐CCTGTAGCCCACGTCGTAGC‐3ʹ, reverse, 5ʹ‐AGCAA TGACTCCAAAGTAGACC‐3ʹ), inducible nitric oxide synthase (iNOS; forward, 5ʹ‐CTAGTGAGTCCCAGTTTTGAAG‐3ʹ, reverse, 5ʹ‐CCCTGGCAGCAGCCATCAGG TA‐3ʹ), CD206 (forward, 5ʹ‐TTCAGCTATTGGACGCGAGG‐3ʹ, reverse, 5ʹ‐GAATCTGACACCCAGCGGAA‐3ʹ), arginase‐1 (Arg‐1; forward, 5ʹ‐CTCCAAGCCAAAGTCCTTAGAG‐3ʹ, reverse, 5ʹ‐AGGAGCTGTCATTAGGGACATC‐3ʹ), and Ym‐2 (forward, 5ʹ‐CAGAACCGTCAGACATTCATTA‐3ʹ, reverse, 5ʹ‐ATGGTCCTTCCAGTAGGTAATA‐3ʹ). PCR products were run on 2% agarose gel and stained with ethidium bromide.

### 2.4 Flow cytometry

Phenotypic analysis of macrophages was performed using flow cytometry. Cells rinsed in phosphate‐buffered saline (PBS; Nacalai Tesque) were recovered from culture plates by scrapping. After incubated with Fc block (CD16/32 antibody; Biolegend) for 5 min, each antibody was added. To assess the differentiation of macrophages, the following antibodies were used: fluorescein isothiocyanate (FITC)‐conjugated anti‐CD11b; phycoerythrin (PE)‐conjugated anti‐F4/80; allophycocyanin (APC)‐conjugated anti‐CD38; Per‐CP‐Cy5.5‐conjugated anti‐CD206 (Biolegend). M1 and M2 macrophages were defined as CD11b^+^F4/80^+^CD38^+^CD206^−^ and CD11b^+^F4/80^+^CD38^−^CD206^+^ cells, respectively^26^ and analyzed using the FACS Canto II Flow Cytometer (BD Biosciences) with FACSDiva software (BD Biosciences).

### 2.5 Mixed lymphocyte reaction

T lymphocytes were isolated from the spleen of the donor (B6 mice) using the Pan T Cell Isolation Kit (Miltenyi Biotec) and used as responder cells. Spleen cells obtained from the recipient (BALB/c mice) were irradiated at 20 Gy and used as stimulator cells. 4 × 10^5^ responder T cells were mixed and cocultured at a ratio of 1:1 with stimulator cells in flat‐bottom 96‐well microtiter plates in a total volume of 0.2 ml complete medium for 5 days at 37°C. To evaluate the effects of M2 macrophages, 4 × 10^5^ M2 macrophages were added directly to the stimulator and responder cells in lower chambers. To evaluate the effect of soluble factors, M2 macrophages were introduced into the transwell inserts (upper chambers). Transwell inserts were placed at 0.5 mm above the well bottom and the pore size of insert membrane was 3.0 μm to facilitate the free diffusion of soluble factors and to prevent M2 macrophages from migrating through. 10 μM bromide oxyuridine (BrdU) was added to the wells 16 h before the end of a 5‐day culture. Cell proliferation of responder cells was measured by BrdU Cell Proliferation Assay Kit (Sigma‐Aldrich). The results were analyzed by measuring the optical density (OD) values. The experiments were performed in triplicates.

### 2.6 HSCT procedure

Mice underwent HSCT as previously described previously.^27^, ^28^ A murine model of HSCT from B6 donors to BALB/c recipients was constructed. Recipient BALB/c mice were lethally irradiated with 7.5 Gy (two fractions) using a particle accelerator. The mice were intravenously injected 5 × 10^6^ BM‐MNCs alone or together with 1 × 10^7^ splenocytes isolated from donor B6 mice on the following day, defined as Day 0. The transplanted mice were monitored daily for condition and weight. Animals were euthanized if they lost more than 40% of their original body weight, if they lost weight rapidly (more than 1 g per day for 2 consecutive days), or if they became morbid. Mice were divided into five groups: (1) TBI group, mice received irradiation only, (2) BM group, mice were injected with BM‐MNCs alone after irradiation, (3) BM + Spl group, mice were injected with BM‐MNCs and splenocytes after irradiation, (4) M1 macrophage group, mice were injected with 5 × 10^6^ M1 macrophages polarized from donor BM‐MNC in addition to BM‐MNCs and splenocytes after irradiation and (5) M2 macrophage group, mice were injected with 5 × 10^6^ M2 macrophages polarized from donor BM‐MNC in addition to BM‐MNCs and splenocytes after irradiation. The survival post‐HSCT was monitored daily. Clinical GVHD was assessed weekly by the scoring system that incorporates five clinical parameters such as fur texture, weight loss, activity, posture, and skin integrity as described previously.^29^ Liver, colon, and skin from transplanted mice were obtained for histopathological examination on Day 14 post‐HSCT.

### 2.7 Histopathology and immunohistochemical analysis

After mice died or were sacrificed, colon, liver, and skin samples were fixed in 4% phosphate‐buffered paraformaldehyde for 24 h, followed by immersion in 70% ETOH overnight and embedded in paraffin. For each organ, 2‐μm‐thick specimens were processed for hematoxylin and eosin (H&E) and immuno‐histological evaluation. One pathologist who specializes in GVHD analyzed the slides in a blinded fashion. Six parameters were scored for colon according to a 0‐ to 5‐point scale.^30^ Seven parameters were scored for the liver according to a 0‐ to 3‐point scale.^30^ Three parameters were scored for the skin according to a 0‐ to 3‐point scale described by Ferrara et al.^31^ For immuno‐histological evaluation, sections were stained with antibodies against mouse F4/80 (1:200, ab6640, Abcam) to identify common macrophage antigens, Mrc‐1 (1:400, ab64693, Abcam) for M2 phenotype macrophage antigens, and GFP (1:1000, ab6673, Abcam) for GFP antigens. For each retrieved sample, five different microscopic fields at 400× magnification for F4/80, Mrc‐1, and GFP count were evaluated. All microscopy images were obtained using a fluorescence microscope (BZ‐X700, Keyence).

### 2.8 Statistical analysis

The statistical analysis was performed using GraphPad Prism version 7.03 for Mac, (GraphPad Software). Data were reported as mean ± standard error of the mean (*SEM*). The statistical significance of differences was determined by Mann–Whitney *U* test. Overall survival was analyzed by the Kaplan–Meier method, and the log‐rank test was used to test the significance of differences. A *p* < .05 was considered statistically significant.

## 3 RESULTS

### 3.1 Induction of M1 and M2 macrophages

**Figure 1:**
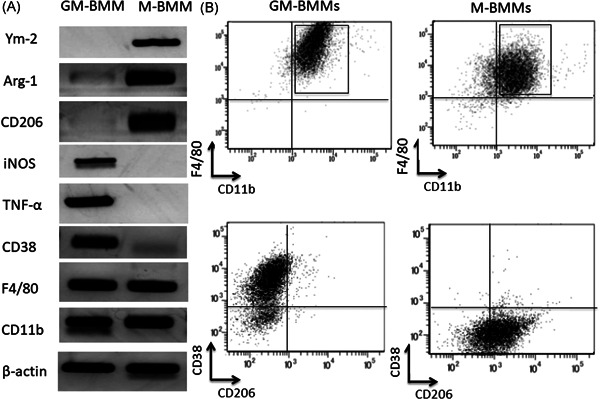
Bone marrow mononuclear cells (BM‐MNCs) are polarized to M1 macrophages and M2 macrophages by stimulation with GM‐CSF and M‐CSF, respectively. (A) Total RNA was isolated from GM‐BMMs and M‐BMMs. The expression of macrophage marker (CD11b and F4/80), M1 macrophage marker (TNF‐α, iNOS, and CD38), and M2 macrophage marker (Ym‐2, Arg‐1, and CD206) were analyzed by RT‐PCR. (B) GM‐BMMs and M‐BMMs were stained with antibodies against CD11b, F4/80, CD38, and CD206, and analyzed by flow cytometry. 92.8% of GM‐BMMs was CD38^+^CD206^−^ and 93.3% of M‐BMMs was CD38^−^CD206^+^

We examined whether BMMs could be differentiated into M1 or M2 macrophages. GM‐CSF, LPS, and IFNγ were used for GM‐CSF‐induced BMMs (GM‐BMMs as M1 macrophages) induction. M‐CSF and IL4 were used for M‐CSF‐induced BMMs (M‐BMMs as M2 macrophages) induction.^18^ Both GM‐BMMs and M‐BMMs expressed common macrophage markers, CD11b and F4/80 (Figure 1A,B). GM‐BMMs were positive for iNOS, TNF‐α, and CD38 (M1 markers) and negative for Ym‐2, Arg‐1, and CD206 (M2 markers) by RT‐PCR (Figure 1A). M‐BMMs were positive for Ym‐2, Arg‐1, and CD206 (M2 markers) and negative for iNOS, TNF‐α, and CD38 (M1 markers) by RT‐PCR (Figure 1A). Surface expressions of M1 and M2 markers were examined by flow cytometry. As shown in Figure 1B, GM‐BMMs were positive for CD38 (M1 marker) and negative for CD206 (M2 marker), whereas M‐BMMs were positive for CD206 and negative for CD38. Since GM‐CSF differentiate BM precursors to DCs, the macrophage fraction may contain DCs.^32^ Flow cytometry analysis for CD11c expression revealed both GM‐BMMs and M‐BMMs were negative for CD11c (data not shown). These results indicate that GM‐BMMs and M‐BMMs have phenotypes of M1 and M2 macrophages, respectively.

### 3.2 Effect of M2 macrophages on GVHD in transplanted mice

**Figure 2:**
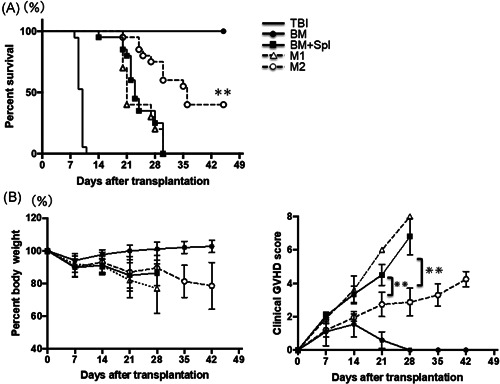
Adoptive transferred M2 macrophages prolong the survival of GVHD mice. Lethally irradiated BALB/c mice received B6 BM‐MNC (5 × 10^6^) alone (filled circle, *n* = 20) or B6 BM‐MNC (5 × 10^6^) + spleen cells (1 × 10^7^). Animals transplanted with adjunctive spleen cells received no other cells (filled squares, *n* = 20), 5 × 10^6^ ex vivo‐polarized M1 macrophages (open triangles, *n* = 10), or 5 × 10^6^ ex vivo‐polarized M2 macrophages (open circles, *n* = 20). (A) Overall survival, (B) body weight loss, and (C) clinical GVHD scores were monitored. Data are the cumulative results of five experiments. Statistical significance was calculated using Log‐rank test; *p < .05, **p < .01. BM, bone marrow; GVHD, graft‐versus‐host disease; MNC, mononuclear cell

To assess the effect of macrophages on GVHD, lethally irradiated BALB/c (H‐2^d^) mice were reconstituted with BM‐MNCs, splenocytes, and BMMs derived from B6 mice (H‐2^b^). All mice transplanted with BM‐MNCs and splenocytes (BM+Spl group) died within 30 days because of severe GVHD (Figure 2A). Chimerism in the BM group was 100% donor type on Day 7 after HSCT (data not shown). All mice transplanted with BM‐MNCs, splenocytes, and M1 macrophages (M1 macrophage group) also died within 30 days because of severe GVHD (Figure 2A). However, mice transplanted with BM‐MNCs, splenocytes, and M2 macrophages (M2 macrophage group) had a significant increase in life span compared with BM + Spl group and M1 macrophage group, with 45% surviving in the M2 macrophage group versus 0% surviving in the BM + Spl group and M1 macrophage group (*p* < .05; Figure 2A). There were no differences in weight change among each group (Figure 2B). Mice in each HSCT group were followed over time for clinical signs of GVHD such as weight loss, dull fur, hunched posture, and diarrhea. Although most mice in the BM + Spl group and M1 macrophage group developed obvious clinical GVHD disease by the end of the 4 weeks after HSCT, clinical GVHD scores of M2 group were significantly lower than those in the BM + Spl group and M1 macrophage group (*p* < .05; Figure 2C). These data clearly showed that cotransplantation of M2 macrophages in addition to BM‐MNCs and splenocytes significantly attenuates GVHD and shows longer survival.

### 3.3 Improvement of pathological GVHD score in liver and colon by injected M2 macrophages

**Figure 3:**
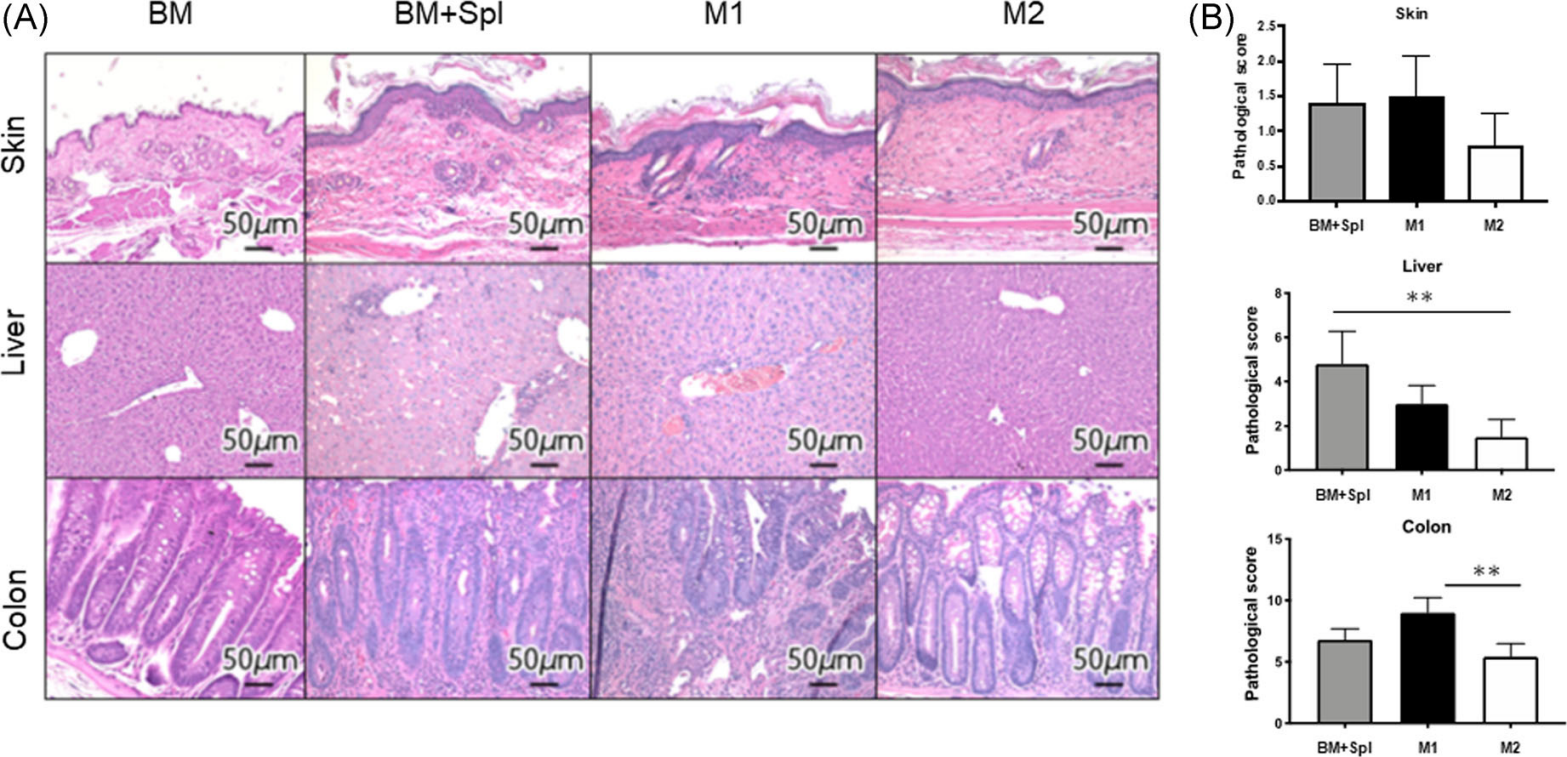
Adoptive transferred M2 macrophages reduce GVHD in liver and colon. (A) Representative images of skin, liver, and colon sections obtained from the indicated groups of mice stained with hematoxylin and eosin on Day 14 after HSCT are shown. (B) Histopathological GVHD scores from mice in each group (n = 4–5/group) were analyzed in a masked fashion by an experienced pathologist in accordance with previously published GVHD histology. Skin (BM + Spl group: 1.4 ± 0.24, M1 macrophage group: 1.5 ± 0.29, M2 macrophage group: 0.8 ± 0.20), Liver (BM + Spl group: 4.8 ± 0.66, M1 macrophage group: 3.0 ± 0.41, M2 macrophage group: 1.5 ± 0.35), Colon (BM + Spl group: 6.8 ± 0.41, M1 macrophage group: 9.0 ± 0.61, M2 macrophage group: 5.4 ± 0.48). Data are presented as the mean ± *SEM*. *p < .05, **p < .01. BM, bone marrow; GVHD, graft‐versus‐host disease; HSCT, hematopoietic stem cell transplantation

To determine whether administration of M2 macrophage attenuated GVHD, histopathological tissue analysis was performed in a masked fashion by an experienced pathologist at Mie University. The sections are taken on Day 14. The representative images of skin, liver, and colon stained with H&E are shown (Figure 3A). When we looked at the liver and colon, we observed severe development of pathological GVHD in the BM + Spl group and M1 macrophage group, but not in the M2 macrophage group. Although no statistical differences were found between groups in terms of the GVHD scoring evaluation in the skin samples, dermal thickening and a paucity of hair follicles, occurring in the BM + Spl group, were not observed in the M2 macrophage group. Regarding the liver, pathological findings of portal inflammation, bile ducts lesions, endothelialitis, and sinusoidal lymphocytosis in the M2 macrophage group were better than GVHD and M1 macrophage groups. Pathological findings of colon in GVHD and M1 macrophage groups showed mucosal ulceration, lamina propria inflammation, crypt loss, crypt epithelial cell apoptosis, crypt regeneration, and villous blunting. These findings were not evident in the M2 macrophage group. Blinded histologic analysis showed significantly reduced GVHD pathology in the liver and colon on Day 14 after transplantation (Figure 3B). These data imply that the administration of M2 macrophage ameliorates the inflammatory response and the pathological progression of GVHD in target organs.

### 3.4 The presence of donor‐derived M2 macrophages in liver and colon of recipients

**Figure 4:**
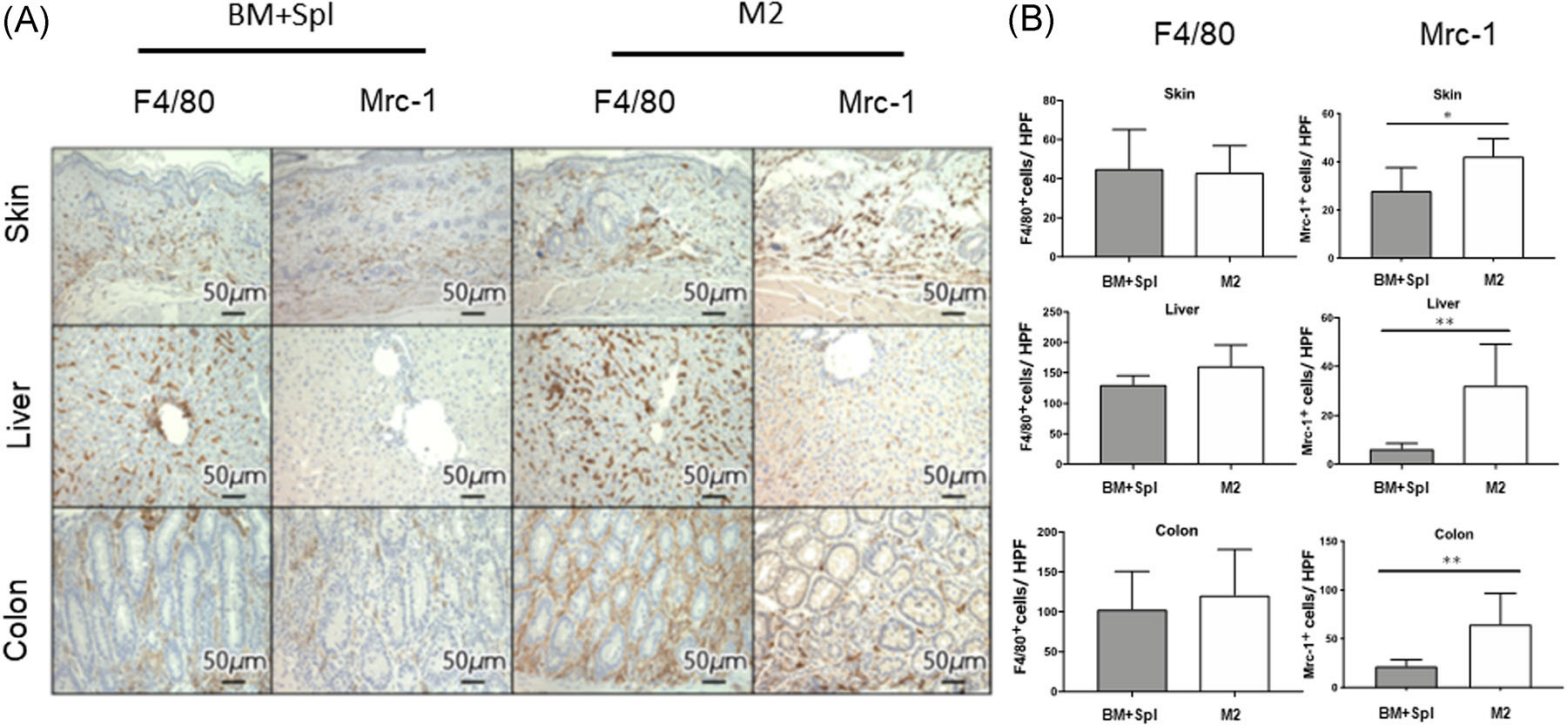
Mrc1+ cells are prevalent in skin, liver, and colon of M2 macrophage group. (A) The representative images stained immunohistochemically with F4/80 and Mrc1 of the skin, liver, and colon in BM + Spl (*n* = 5) and M2 group (*n* = 5) are shown. (B) The number of F4/80 and Mrc‐1 positive cells were counted in each organ from BM + Spl group mice (filled bars *n* = 5) and M2 group mice (open bars *n* = 5) by an experienced pathologist. Data are presented as the mean ± *SEM*. Skin‐F4/80 (BM + Spl group: 45 ± 8.9, M2 macrophage group: 43.2 ± 6.1), Liver‐F4/80 (BM + Spl group: 131.3 ± 6.5, M2 macrophage group: 160.9 ± 15.5), Colon‐F4/80 (BM + Spl group: 103.4 ± 21.0, M2 macrophage group: 121.1 ± 25.4), Skin‐Mrc‐1 (BM + Spl group: 28.0 ± 4.3, M2 macrophage group: 42.3 ± 3.3), Liver‐Mrc‐1 (BM + Spl group: 6.3 ± 1.0, M2 macrophage group: 32.4 ± 7.4), Colon‐Mrc‐1 (BM + Spl group: 22.0 ± 2.8, M2 macrophage group: 64.9 ± 14.2 per 400× high‐performance field). *p < .05, **p < .01. BM, bone marrow

**Figure 5:**
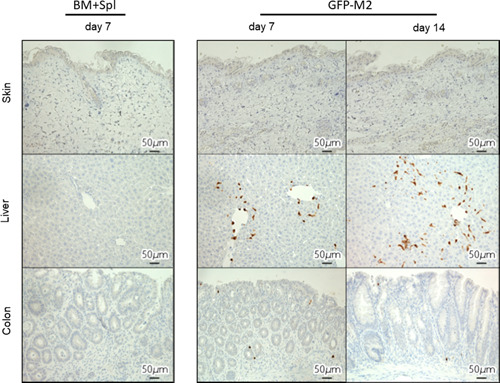
Donor BM‐derived M2 macrophages migrate to the site of GVHD. B6 BM‐MNCs (5 × 10^6^) and spleen cells (1 × 10^7^) were injected to recipient BALB/c mice with or without ex vivo‐generated M2 macrophages (5 × 10^6^) derived from B6‐GFP mice. Skin, liver, and colon are obtained from the indicated group of mice on Day 7 and Day 14, and stained immunohistochemically with GFP. BM, bone marrow; GVHD, graft‐versus‐host disease; MNC, mononuclear cell

To evaluate the number of macrophages to target organs of GVHD, immunohistochemical analysis of F4/80 (a pan macrophage marker) and Mrc‐1 (an M2 macrophage marker) expression in skin, liver, and colon was performed. The number of F4/80 positive macrophages was no difference between the BM + Spl group and the M2 macrophage group (Figure 4A,B, left column). However, macrophages in skin, liver, and colon of M2 group showed significantly more Mrc‐1 positive than those of BM + Spl group (Figures 4A,B, right column). To confirm whether Mrc‐1 positive M2 macrophages in target organs were originated from donor, M2 macrophages derived from green fluorescent protein transgenic B6 (B6‐GFP) mice were transplanted to lethally irradiated BALB/c mice in addition to wild type B6 derived BM‐MNCs and splenocytes. Immunohistochemical analysis of GFP expression revealed that GFP positive M2 macrophage was localized in liver and colon (Figure 5, middle and lower lines). These data showed that adoptively transferred M2 macrophages derived from donor BM can migrate to target organs of GVHD. Interestingly, the macrophages can survive in the target organs for 2 weeks.

### 3.5 The inhibitory effect of M2 macrophage on T cell proliferation in allogeneic reaction

**Figure 6:**
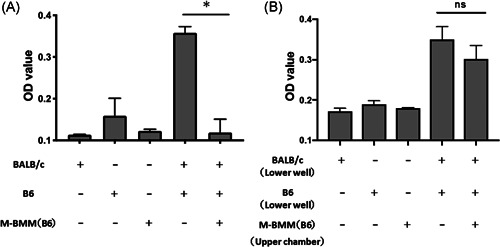
M2 macrophages inhibit proliferation of T lymphocytes by cell‐to‐cell contact. (A) Irradiated spleen cells from the BALB/c mice were used to stimulate B6 mice‐derived T lymphocytes. The ability of BALB/c splenocytes to stimulate B6 T lymphocytes was determined by the bromide‐uridine (BrdU) cell proliferation assay kit. M2 macrophages induced from B6 BM‐MNCs were cocultured with B6 T lymphocytes and irradiated BALB/c splenocytes. (B) M2 macrophages induced from B6 BM‐MNCs were cultured in transwell inserts to avoid direct contact to cocultured T cells and splenocytes. Results are representative of three independent experiments. Data are presented as the mean ± *SEM*. **p < .01. BM, bone marrow; MNC, mononuclear cell

To determine whether donor BM‐derived M2 macrophages were anergic, donor (B6) T lymphocytes were cultured in presence of irradiated recipient (BALB/c) splenocytes with or without donor (B6) derived M2 macrophages. Cell proliferation of donor T lymphocytes was assessed by BrdU incorporation into the nuclear DNA. The mean OD value of the GVHD control group (B6 T lymphocytes + irradiated BALB/c splenocytes) was 0.36 ± 0.01, while the mean OD value of M2 macrophage group (B6 T lymphocytes + irradiated BALB/c splenocytes + B6 derived M2 macrophages) was 0.12 ± 0.02 (*p* < .05; Figure 6A). This indicated that donor‐derived M2 macrophages have suppressive effect on the proliferation of donor T cells.

To further address whether donor M2 macrophages modulate T cell proliferation by soluble factors or in cell contact‐dependent manner, a transwell coculture system was used to prevent cell‐to‐cell contact between macrophages and T lymphocytes. B6‐derived M2 macrophages in the upper chamber were cocultured with B6 T cells + irradiated BALB/c splenocytes. As shown in Figure 6B, no statistically significant changes in B6 T cell proliferation were observed in transwell cultures. The mean OD value of the control group (B6 T cells + irradiated BALB/c splenocytes in lower wells) was 0.35 ± 0.02, while the mean OD value of the M2 macrophage in the upper chamber group (B6 T cells + irradiated BALB/c splenocytes in lower wells + M2 macrophages in upper chambers) was 0.30 ± 0.02 (*p* = .25) (Figure 6B). These data suggested that M2 macrophages suppressed the proliferation of donor‐derived T lymphocytes by cell‐to‐cell contact.

## 4 DISCUSSION

Our study revealed the important role of donor BM‐derived M2 macrophages in modulating GVHD severity after HSCT. In this study, we demonstrate that donor BMMs polarized to an M2 phenotype ex vivo suppress proliferation of donor alloreactive T cells by cell‐to‐cell contact. We also establish that the administration of donor‐derived M2 macrophages improves GVHD in transplanted mice through the M2 macrophage migration into the GVHD target organs. Surprisingly, the donor BM‐derived M2 macrophages can survive in the target organs of the recipient mice for 2 weeks.

Our data demonstrate that M2 macrophages improve GVHD through inhibiting the expansion of alloreactive T lymphocytes in a cell contact‐dependent manner. However, the mechanism through which M2 macrophages control immune responses has not been identified in our current study. Multiple mechanisms have been reported to relate to immunosuppressive capacity of M2 macrophages. M2 macrophages suppress T cell responses by producing Arg‐1 enzyme that depletes l‐arginine, which is the substrate of iNOS.^33^ In our study, M2 macrophages expressed higher *Arg‐1* than M1 macrophages. Furthermore, M2 macrophages suppressed GVHD effectively than M1 macrophages. Therefore, we believe that donor BM‐derived M2 macrophages have stronger anti‐alloreactive effects than M1 macrophages. In our study, a single administration of M2 macrophages increased the survival of transplanted mice in the M2 macrophage group. Therefore, a higher proportion of mice could survive by repeat regular injections of M2 macrophages. GFP+ macrophages that were transferred as M2 macrophages were found in skin, liver, and colon at 2 weeks after HSCT. It is important to investigate the fate of the infused M2 macrophages. In the next study, we investigate whether the infused M2 macrophages still have M2 phenotype after HSCT and whether the infused M2 macrophages would alter endogenous macrophage differentiation and infiltration to cause GVHD.

Recently, Holtan et al.^34^ showed that M2 macrophages accumulated in colonic mucosa during steroid‐refractory acute GVHD and their presence may indicate incomplete tissue repair. In their report, it is not known whether M2 macrophages accumulated in colon are donor‐derived or host‐derived. Although ex vivo‐polarized donor M2 macrophages can suppress GVHD, recipient‐derived M2 macrophages may be unable to inhibit alloreaction. Further functional studies of the mucosal cells will be required to clarify the role of M2 macrophages in GVHD.

It has not been clear whether the regulation of alloreactive T lymphocytes by M2 macrophages depends on soluble factors or direct cell‐to‐cell interaction. Although our data strongly suggest that the direct interaction between T lymphocytes and M2 macrophages is involved in the immunoregulation of alloreactive T lymphocytes, the specific molecular mechanisms are unclear. Previous studies have reported that M2 macrophages inhibit the proliferation of T lymphocytes by programmed death‐ligand 2 (PD‐L2).^35^ Activation of Stat6 in response to IL‐4 is necessary for macrophage PD‐L2 expression.^35^ Furthermore, PD‐L1 expression was upregulated on M2‐polarized macrophages and PD‐L1‐positive macrophages reduced cytotoxicity and proliferation of T lymphocytes.^36^ Therefore, it is worth to further confirm whether surface PD‐L1 and/or PD‐L2 expression on M2 macrophages is necessary to inhibit alloreactive T cell function.

Activated macrophages play important roles in inflammation, tissue homeostasis, and disease pathogenesis. Since various mediators have been used to generate activated macrophages, Murray et al recommended to describe stimulation scenarios and adopt a nomenclature linked to the activation standards.^37^ According to their nomenclature, GM‐BMMs should be macrophages (GM‐CSF(GC)+LPS+IFNγ) and M‐BMMs should be macrophages (IL‐4). To generate mouse DCs, BM‐MNCs are usually cultured with GM‐CSF.^38^ The product from GM‐CSF culture is heterogeneous and contains granulocytes and macrophages as well as DCs.^32^ Therefore, GM‐BMMs fraction in our study may contain DCs and neutrophils in addition to macrophages. To precisely characterize GM‐BMMs and M‐BMMs in this study, the expression of DC marker CD11c was analyzed.

In this study, the kinetics of macrophage recruitment to GVHD target organs after HSCT were investigated in lethally irradiated BALB/c mice who were transplanted with B6 graft. The B6 into BALB/c strain combination has been well‐documented to result in lethality from acute GVHD.^27^, ^28^ However, many current GVHD studies use additional strain combinations to validate the GVHD effects. By using sclerodermatous chronic GVHD model, Alexander et al reported that donor macrophage transfer can result in increased cutaneous chronic GVHD.^39^ Therefore, careful studies using other GVHD models need to be performed on M1 versus M2 macrophage effects.

In conclusion, this is the first study to demonstrate that donor BM‐derived M2 macrophages have a great potential in GVHD treatment. The infused M2 macrophages sufficiently migrate to the sites of GVHD and have been shown to suppress T lymphocyte proliferation by cell‐to‐cell contact. Hence, our findings suggest novel therapeutic strategies for GVHD based on the use of donor‐derived M2 macrophages.

## AUTHOR CONTRIBUTIONS

Ryo Hanaki carried out most of the RT‐PCR, flow cytometer analyses, animal studies, the statistical analysis, and drafted the manuscript. Hidemi Toyoda participated in the design of the study and performed the revised manuscript. Mari Morimoto, Daisuke Nakato, Takahiro Ito, Kaori Niwa, and Keishiro Amano carried out a part of RT‐PCR and cell culture. Ryotaro Hashizume participated in histopathology and immunohistochemical analysis. Isao Tawara and Masahiro Hirayama participated in revising manuscript. Masahiro Hirayama, Isao Tawara, Shotaro Iwamoto, and Hidemi Toyoda participated in the conception of the study. Masahiro Hirayama conceived of the study, and participated in its design and coordination, and helped to draft the manuscript. All authors read and approved the final manuscript.
